# Copper-Containing Alloy as Immunoregulatory Material in Bone Regeneration via Mitochondrial Oxidative Stress

**DOI:** 10.3389/fbioe.2020.620629

**Published:** 2021-01-14

**Authors:** Daorong Xu, Jikun Qian, Xin Guan, Ling Ren, Kaifan Yang, Xuan Huang, Shuyuan Zhang, Yu Chai, Xiaohu Wu, Hangtian Wu, Xianrong Zhang, Ke Yang, Bin Yu

**Affiliations:** ^1^Division of Orthopedic Surgery, Department of Orthopedics, Nanfang Hospital, Southern Medical University, Guangzhou, China; ^2^Guangdong Provincial Key Laboratory of Bone and Cartilage Regenerative Medicine, Nanfang Hospital, Southern Medical University, Guangzhou, China; ^3^Institute of Metal Research, Chinese Academy of Sciences, Shenyang, China; ^4^State Key Laboratory of Organ Failure Research, Guangdong Provincial Key Laboratory of Viral Hepatitis Research, Department of Infectious Diseases, Nanfang Hospital, Southern Medical University, Guangzhou, China

**Keywords:** 316-5Cu stainless steel, type H vessel, M2a macrophage, PDGF-BB, immunoregulation

## Abstract

In the mammalian skeletal system, osteogenesis and angiogenesis are closely linked by type H vessels during bone regeneration and repair. Our previous studies confirmed the promotion of these processes by copper-containing metal (CCM) *in vitro* and *in vivo*. However, whether and how the coupling of angiogenesis and osteogenesis participates in the promotion of bone regeneration by CCM *in vivo* is unknown. In this study, M2a macrophages but not M2c macrophages were shown to be immunoregulated by CCM. A CCM, 316L−5Cu, was applied to drilling hole injuries of the tibia of C57/6 mice for comparison. We observed advanced formation of cortical bone and type H vessels beneath the new bone in the 316L−5Cu group 14 and 21 days postinjury. Moreover, the recruitment of CD206-positive M2a macrophages, which are regarded as the primary source of platelet-derived growth factor type BB (PDGF-BB), was significantly promoted at the injury site at days 14 and 21. Under the stimulation of CCM, mitochondria-derived reactive oxygen species were also found to be upregulated in CD206^hi^ M2a macrophages *in vitro*, and this upregulation was correlated with the expression of PDGF-BB. In conclusion, our results indicate that CCM promotes the evolution of callus through the generation of type H vessels during the process of bone repair by upregulating the expression of PDGF-BB derived from M2a macrophages.

**Graphical Abstract:**
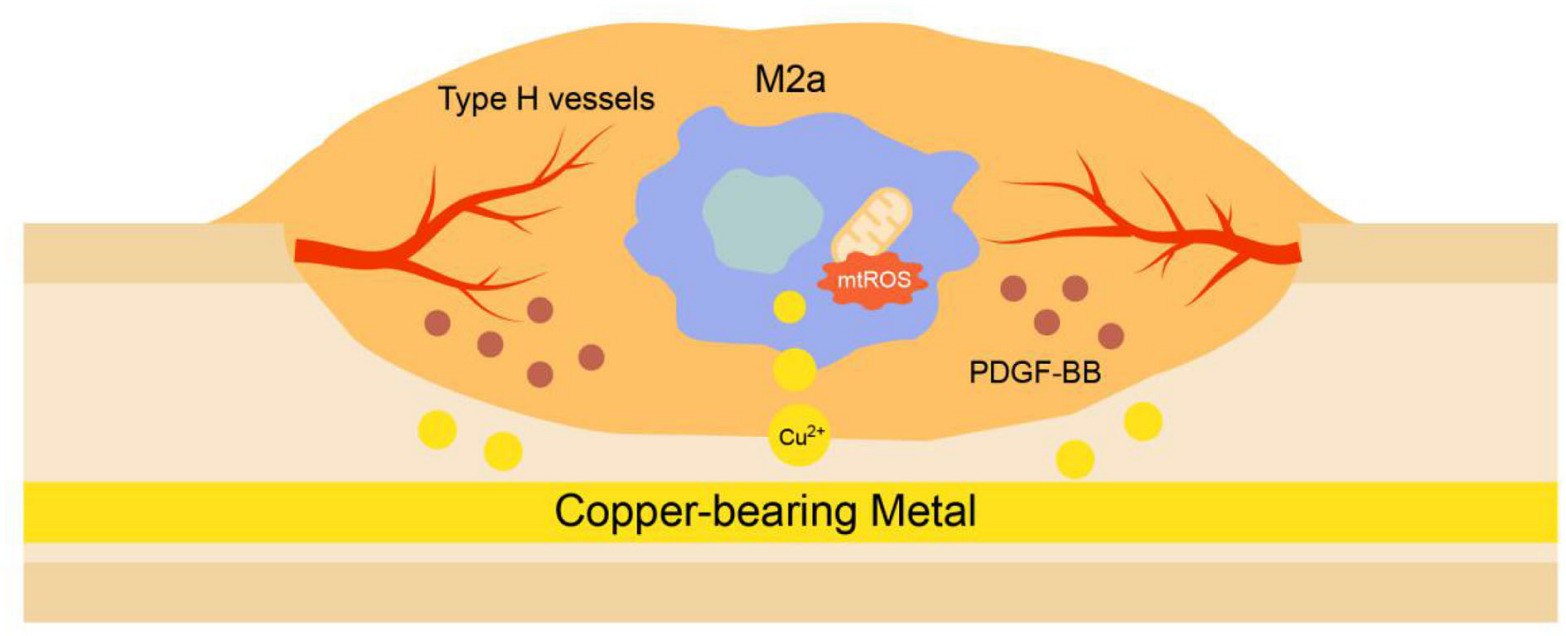
316L−5Cu stainless steel promoted the evolution of callus through the generation of type H vessels in the process of bone repair by upregulating the expression of PDGF-BB derived from M2a macrophages, which contributes to the expression of mitochondria-derived reactive oxygen species due to copper stress.

## Highlights:

- Copper-containing metal promoted the evolution of callus through the generation of type H vessels in the process of bone repair.
- CD206+ M2a macrophage-derived PDGF-BB were elevated by copper-containing metal in callus beneath the newly formed cortical bone.
- The coupling of osteogenesis and angiogenesis was linked by immunoregulation of copper-containing biomaterials.

## Introduction

Bone fracture is an increasingly common medical incident that results from both traumatic injury and disease-related bone fragility. Even when adequate bone repair is achieved within the expected time frame, treating fractures is costly and can be protracted in the 10–20% of cases in which healing is delayed or fails (Parker et al., 2007; Hernandez et al., 2012). The limited treatment options for accelerating fracture repair and sustaining peak bone mass throughout life represent a growing clinical problem. Current biological treatments (for example, teriparatide and strontium ranelate) are modestly effective or not broadly applicable, creating a treatment gap in the management of fractures and osteoporosis (Wu et al., 2013).

The intimate spatial and temporal link between osteogenesis and angiogenesis, termed angiogenic–osteogenic coupling, can recruit both endothelial and osteoblast precursor cells to the locus (Zheng et al., 2018; Peng et al., 2020). A recent study by Romeo et al. (2019) reported that type H vessels play a crucial part in promoting the conversion of cartilage matrix to bone tissue during bone development and regeneration. Platelet-derived growth factor type BB (PDGF-BB), which is derived from macrophages, contributes to the generation of type H vessels in the first stages of these processes. It was also noted in a recent study that emcn^hi^CD31^hi^ endothelium was present during endochondral bone formation in the osteotomy gap (Stefanowski et al., 2019). Moreover, administration of recombinant SLIT3, which is derived from osteoblasts and promotes formation of type H vessels, effectively accelerated bone fracture healing in a mouse bone fracture model (Xu et al., 2018). Thus, it was proposed that fracture healing could be enhanced by promoting type H vessel angiogenesis, providing a potential therapeutic approach, particularly for cases of delayed union or non-union.

The most popular biomaterials used in managing bone fractures are metals including titanium alloy and stainless steel, which promote bone repair and have specific effects depending on their components (Spiller et al., 2014; Inzana et al., 2016). Magnesium, a transition metal element that contributes to the biological activity regulated by neurotransmitters in bone, is favored for modification of the composition of orthopedic medical metals (Zhang et al., 2016). Another transition metal, copper, which is known to be an antibacterial agent, is noted for its promotion of osteogenesis and angiogenesis (Ren et al., 2012, 2015; Ryan et al., 2019). Our previous studies showed that copper-containing stainless steel promoted fracture healing by accelerating the process of callus evolution in a fracture model (Wang et al., 2015). In the research by Chen et al. (2020), focusing on the underlying mechanism, copper promoted the migration of bone marrow mesenchymal stem cells via Rnd3-dependent cytoskeleton remodeling; however, there was no involvement of angiogenesis. Hence, exploring whether copper participates in the coupling of osteogenesis and angiogenesis in bone regeneration could enhance our understanding of this mechanism.

The immune system protects the body by eradicating pathogenic microorganisms; however, its additional physiological and pathological roles in a variety of biological systems, including the musculoskeletal system, are being gradually discovered. Xie et al. (2014) demonstrated that PDGF-BB enhanced the formation of type H vessels and bone during bone modeling and remodeling. They showed that macrophages/non-resorbing osteoclast lineage cells are the main sources of PDGF-BB in bone marrow, which further recruits both endothelial and osteoblast precursor cells, thereby coupling angiogenesis with osteogenesis. More specifically, Spiller et al. (2014) found that M2a macrophages marked with CD206^high^ secreted the most PDGF-BB *in vitro*, compared with M1 and M2c macrophages. The present study shows that copper-containing metal (CCM) promotes bone repair in a drilling hole injury model through enhancing the generation of type H vessels via recruiting and activating infiltrating M2a macrophages in callus.

## Methods and Materials

### Copper-Containing Stainless Steel

Copper-containing stainless steel was designed and fabricated by the addition of the appropriate amount of copper to medical stainless steel. The experimental materials used in this study included a previously reported 316L−5Cu stainless steel with a nominal chemical composition (wt%) of Cr 19, Ni 13, Mo 3.5, Cu 4.5, and Fe in balance, which was obtained by vacuum induction melting. Purchased medical-grade 316L stainless steel was used for comparison (Ren et al., 2012, 2015). Samples were cut from forged bars into disks of 1 mm thickness and small disks of 5 mm diameter and 1 mm thickness for use in the *in vitro* experiments. Intramedullary nails, which were used to create tibia injury in C57 mice *in vivo*, were processed by cold-drawing 316L−5Cu stainless steel to 0.45 mm diameter and then cut into 9-mm lengths. All the experimental steels with single austenitic structures were solution treated at 1,050°C for 0.5 h, followed by water quenching, and then aged at 700°C for 6 h to achieve Cu-rich precipitation in the 316L−5Cu stainless steel; this was expected to promote the release of Cu ions from the surface of the steel. All the samples were ground with SiC sand papers up to grade 2000#, soaked in absolute ethanol, cleaned with ultrasonic and deionized water, and finally sterilized at 121°C prior to use in experiments.

### Preparation of Extracted Medium and Polarized Macrophage Culture

Each steel sample was placed in one well of a 24-well culture plate. Sugar-rich Dulbecco's modified Eagle's medium (DMEM) was dropped onto the samples (1 ml medium per 3 cm^2^ surface area of experimental material), followed by incubation at 4°C for 72 h. The media cocultured with the steel samples were extracted and collected.

RAW264.7 cells were purchased from Cyagen Biosciences (China). High-glucose DMEM (Life Technologies, USA) containing 1% (v/v) penicillin/streptomycin (Thermo, USA) and 10% fetal bovine serum (Thermo, USA) was used for culture of RAW cells. RAW cells were incubated at 37°C with 5% CO_2_. Additional drugs, namely, Mito-TEMPO (a mitochondria-targeted ROS antagonist, 50 nM, Sigma), were added to the culture medium for 60 min before polarization.

Polarization was started by changing the culture medium for complete medium, 316L−5Cu extracted medium, or 316L extracted medium, supplemented with the following cytokines: 40 ng/ml recombinant murine interleukin (IL)-4 (PeproTech, 214-14) and 20 ng/ml recombinant murine IL-13 (PeproTech, 210-13) for M2a; and 40 ng/ml recombinant murine IL-10 (PeproTech, 210-10) for M2c. After 48 h of polarization, cells and culture supernatants were collected for further experiments.

### Western Blotting

Total protein was extracted from RAW 264.7 cells using the same procedures as described in detail elsewhere (Santa et al., 2016). Briefly, raw cells were lysed using radioimmunoprecipitation assay (RIPA) lysis buffer (Thermo, 89901) containing proteinase inhibitors and phosphatase inhibitors (KeyGEN BioTECH, KGP250). The protein concentrations were determined using a bicinchoninic acid (BCA) protein assay kit (Thermo Fisher Scientific, 23,225). Subsequently, the phenol–ethanol supernatant was mixed with isopropanol to isolate proteins. Equal amounts of protein and supernatants were separated by 12.5 or 15% sodium dodecyl sulfate polyacrylamide gel electrophoresis and transferred to 0.45 or 0.20 μm-pore polyvinylidene fluoride membranes (Merck Millipore, IPVH00010 or ISEQ00005). Membranes were incubated overnight with antibodies against CD163 (Abcam, ab182422), CD206 (Abcam, ab64693), PDGF-BB (Santa Cruz, sc365805), MMP9 (Abcam, ab38898), and β-actin (Cell Signaling echnology, 2118). The membranes were then incubated with peroxidase-conjugated goat antirabbit immunoglobulin G (IgG) (h + l) secondary antibody (1:5,000) for 1 h. Protein signals were detected using an enhanced chemiluminescence kit (Cat. WBKLS0500, Millipore), and Western blot bands were examined and analyzed with a chemiluminescence instrument (Guangzhou Ewell Bio Technology Co. Ltd., China).

### Animals and Surgical Procedure

Male C57BL/6 mice (10–12 weeks old) were housed at a controlled temperature (22 ± 1°C) with a light–dark cycle (7:00 am to 7:00 pm) and allowed food and water *ad libitum*. They were randomly divided into two evenly sized groups: a control (316L) group and a 316L−5Cu group. Mice were anesthetized, and hair was removed from the left hind limb. An incision was made on the skin over the medial aspect of the proximal tibia. Soft tissue was cleared from the distal end of the tibial crest, and a hole (0.8 mm in diameter) that penetrated through both the medial cortices and the intervening medulla was created in the bone using a 21-gauge needle. A stainless steel pin (316L or 316L−5Cu) with length of 8 mm and diameter of 0.45 mm was inserted into the tibial medullary cavity from the proximal tibia following skin closure.

The study design and procedures were entirely in accordance with the US National Research Council (2011). This study was approved by the Animal Ethics Committee of Nanfang Hospital, Southern Medical University.

### Microcomputed Tomography

After being fixed in 10% formalin solution for 12 h at 4°C, microcomputed tomography (μCT) scanning was performed (Scanco Medical, AG, Switzerland) with X-ray energy of 55 kvp and a current of 145 mA, voxel size of 9 μm, and an integration time of 400 ms. The scanned images were reconstructed with NRecon (v1.6), and the data were analyzed using CTAn (v1.9) and three-dimensional model visualization software (μCTVol v2.0). A sequence of images within the bone defect were chosen for analysis. Three-dimensional histomorphometric analysis was performed using longitudinal images of the tibia. The region of interest was set as the cylindrical region bordered by the defect edge. The three-dimensional structural parameter of bone volume to tissue volume ratio was analyzed.

### Histological, Immunohistochemistry, and Immunofluorescence Analysis

Mice (*n* = 12 per group) were sacrificed, and tibia specimens were harvested at days 7, 14, and 21 postinjury for observation of the histological and histochemical alterations after injury. After being fixed in 10% formalin solution for 12 h at 4°C, the specimens were decalcified in 10% ethylenediaminetetraacetic acid (EDTA) (Sigma) solution for 4.5 days. Two incisions, parallel with the major axis of the drilling hole, were made on the ventral and dorsal sides of the tibia, respectively. At the crevice of the incisions, the bone tissue surrounding the implant was carefully divided into two parts and forced apart from the implant surface, minimizing the damage to the interface tissues.

Samples were immersed in 30% (w/v) sucrose in 0.1 M phosphate buffer (pH 7.3) overnight at 4°C, embedded in paraffin (*n* = 6) and optimal cutting temperature compound (*n* = 6, Sakura Finetek), and then cut into 6 μm thick sections longitudinally for hematoxylin and eosin (H&E) and immunohistochemical staining, and 10 μm thick sections in a cryostat (Leica CM1800; Heidelberg, Germany) for immunofluorescence staining.

For H&E staining, some of the sections obtained previously were preheated in an air oven at 60°C, followed by deparaffinization and rehydration in xylene and ethanol solutions (reducing concentrations of 100 to 70%). The sections were then successively soaked in H&E dyes. After dehydration with graded alcohol, histological observation was performed using a microscope (BX63, Olympus, Tokyo, Japan), and the fracture healing process was evaluated.

Immunohistochemical staining was performed on the deparaffinized and rehydrated sections as described previously, with specific primary antibody (rabbit anti-CD206; ab64693, Abcam 1:200, UK). All the sections were counterstained using Mayer's hematoxylin (Sigma-Aldrich) and mounted using a permanent mounting medium (Thermo Fisher Scientific, Waltham, MA, USA). Quantification of positive cell numbers within the injury site was carried out on 4 μm serial sections stained for CD206 expression. Three representative images (×40 magnification) were taken within the intramedullar injury zone for each sample at two sectional depths at least 50 μm apart. The number of CD206+ cells was quantified by counting each positively stained cell in each field of view.

Frozen sections embedded in optimal cutting temperature (OCT) were processed for immunofluorescence staining of Emcn, CD31,F4/80, CD163, CD206, and PDGF-BB. Sections were incubated in blocking buffer (10% goat serum in phosphate-buffered saline; PBS) for 1 h at room temperature and incubated with primary antibodies overnight at 4°C. The following primary antibodies were used for immunostaining: rat antiendomucin (Emcn, SC-65495, Santa Cruz, 1:50, US), rabbit anti-CD31 (ab222783, Abcam, 1:50, UK), rat anti-F4/80 (71299,Cell Signaling Technology, 1:500, USA), rabbit anti-CD206 (ab64693, Abcam 1:200, UK), rabbit anti-CD163 (ab182422, Abcam, 1:200, UK), and mouse anti-PDGF-BB (sc-365805, Santa Cruz, 1:50, USA). Sections were washed three times in PBS and then incubated with secondary antibodies at room temperature for 1 h. The following secondary antibodies were used for immunostaining: Alexa Fluor 594-conjugated goat antirabbit IgG (8889, Cell Signaling Technology, USA), Alexa Fluor 488-conjugated goat antirat IgG (4416, Cell Signaling Technology, USA), and 594-conjugated goat antimouse (HA1112, HuaBio, China). Nuclei were counterstained with 4′,6-diamidino-2-phenylindole (DAPI) (S2110, Solarbio, China). All the sections were observed under a BX63 microscope (Olympus, Tokyo, Japan).

Quantitative analysis was carried out using three representative images (×40 magnification) taken within the intramedullar injury zone for each specimen at three sectional depths at least 50 μm apart. The number of positive cells was quantified by counting each positively stained cell in each field of view.

### Mitochondrial Potential Detection Using Mitotracker Green and Mitotracker Orange

By combining both Mitotracker Green (MTG) and Mitotracker Orange (MTO) (Invitrogen, CA, USA), the relative mitochondrial potential with its mitochondrial mass as baseline can be obtained (Agnello et al., 2008). Polarized and pretreated RAW264.7 cells were seeded in six-well chamber slides and grown to 50–70% confluence. After washing twice with warm 1× PBS, cells were stained with 100 μM MTG, 500 μM MTO, and Hoechst 33324 (HuaBio, China) in DPBS + Ca/Mg/glucose to maintain their normal metabolic state. Cells were then incubated for 45 min at 37°C under standard culture conditions. The u-slide was then transferred to a confocal microscopic station with a 37°C heated chamber supplied with 5% CO_2_ for live cell imaging. Tile scan images were captured randomly using the same TCS-SP2 confocal microscopy system (Leica Microsystem, Nussloch GmbH, Germany) with sequential detection for both stains. The fluorescence intensities of MTO (excitation/emission, 554/576 nm) and MTG (excitation/emission, 490/516 nm) were measured. For five samples from each group, a total of 20 different microscopic fields per sample, containing comparable numbers of cells, were integrated to obtain fluorescence mean pixel intensity values. The ratio of MTO/MTG intensities was then calculated and denoted the mean index of MTO/MTG, reflecting the relative mitochondrial potential.

### Mitochondrial ROS Assay

To determine mitochondrial superoxide production, polarized and pretreated RAW 264.7 cells were incubated for 30 min with 100 μM MitoSOX dye (Thermo Fisher Scientific, M36008) and Hoechst 33324 diluted in cell imaging solution (10 mM HEPES, 1 mg/ml bovine serum albumin, 1 mg/ml glucose, 1 mM MgCl_2_, and 1.8 mM CaCl_2_ in PBS) at 37°C in a humidified atmosphere containing 5% CO_2_. Then, cells were washed with PBS. The fluorescence was read using a SpectraMax i3x microplate reader at 510/580 nm, and the cell number in each well was determined using a SpectraMax MiniMax 300 imaging cytometer. Fluorescence values were first normalized to the cell number in each well; then, all the conditions for each genotype were normalized to the non-treated control group.

### Statistical Analysis

Statistically significant differences were determined using unpaired *t*-tests with one- or two-tailed distributions using PRISM 7 (GraphPad software, La Jolla, CA, USA). A value of *p* < 0.05 was deemed statistically significant. In all cases, data are represented as mean ± standard error of the mean (SEM).

## Results

### CCM Promotes the Expression of PDGF-BB in M2a Macrophages

**Figure 1:**
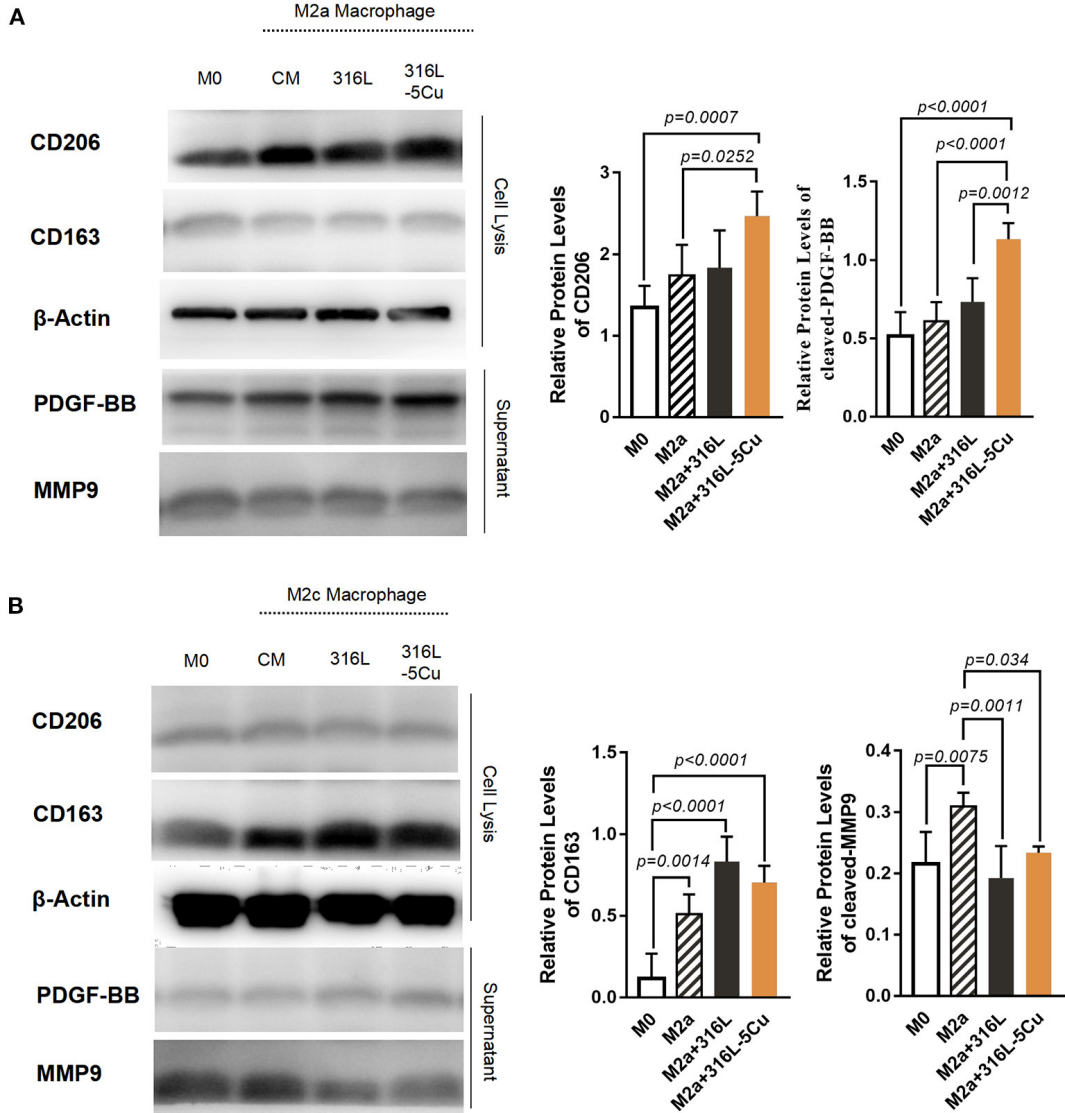
Copper-containing metal (CCM) promoted the expression of platelet-derived growth factor type BB (PDGF-BB) in M2a macrophages. Western blotting was performed to examine the expression levels of CD206, CD163, PDGF-BB, and MMP9 in the following macrophages: M0, M2a, M2c, M2a with 316L, M2c with 316L, M2a with 316L−5Cu, and M2c with 316L−5Cu. β-Actin from cell lysis was used as a loading control. **(A)** Representative images of Western blots and quantification of relative protein levels of CD206 and PDGF-BB in M2a macrophages. **(B)** Representative images of Western blots and quantification of relative protein levels of CD163 and MMP9 in M2c macrophages. Data were statistically analyzed by Student's *t*-test. Results are presented as mean ± SEM. *n* = 5.

M2a and M2c, two subtypes of macrophages, are known to be closely associated with angiogenesis, linked by IL4, IL13, and IL10 (Wu et al., 2013; Spiller et al., 2014). To identify the subtype with the major role under CCM stimulation, Western blotting was performed on M2 macrophages *in vitro*. Upon copper stimulation, M2a cells with high expression of CD206 and M2c cells with higher CD163 expression were detected. The expression levels of PDGF-BB and CD206 were higher in M2a macrophages stimulated by 316L−5Cu extract (Figure 1A), and CD206 and CD163 were identified in this distinct type of macrophage (Figures 1A,B). Notably, the expression levels of MMP9 and CD163 showed no difference between the 316L and 316L−5Cu groups (Figure 1B), indicating that M2a macrophages predominantly promoted the expression of PDGF-BB under the stimulation of 316L–Cu extract.

### The Callus Formation Process of Bone Repair Is Accelerated by CCM

**Figure 2:**
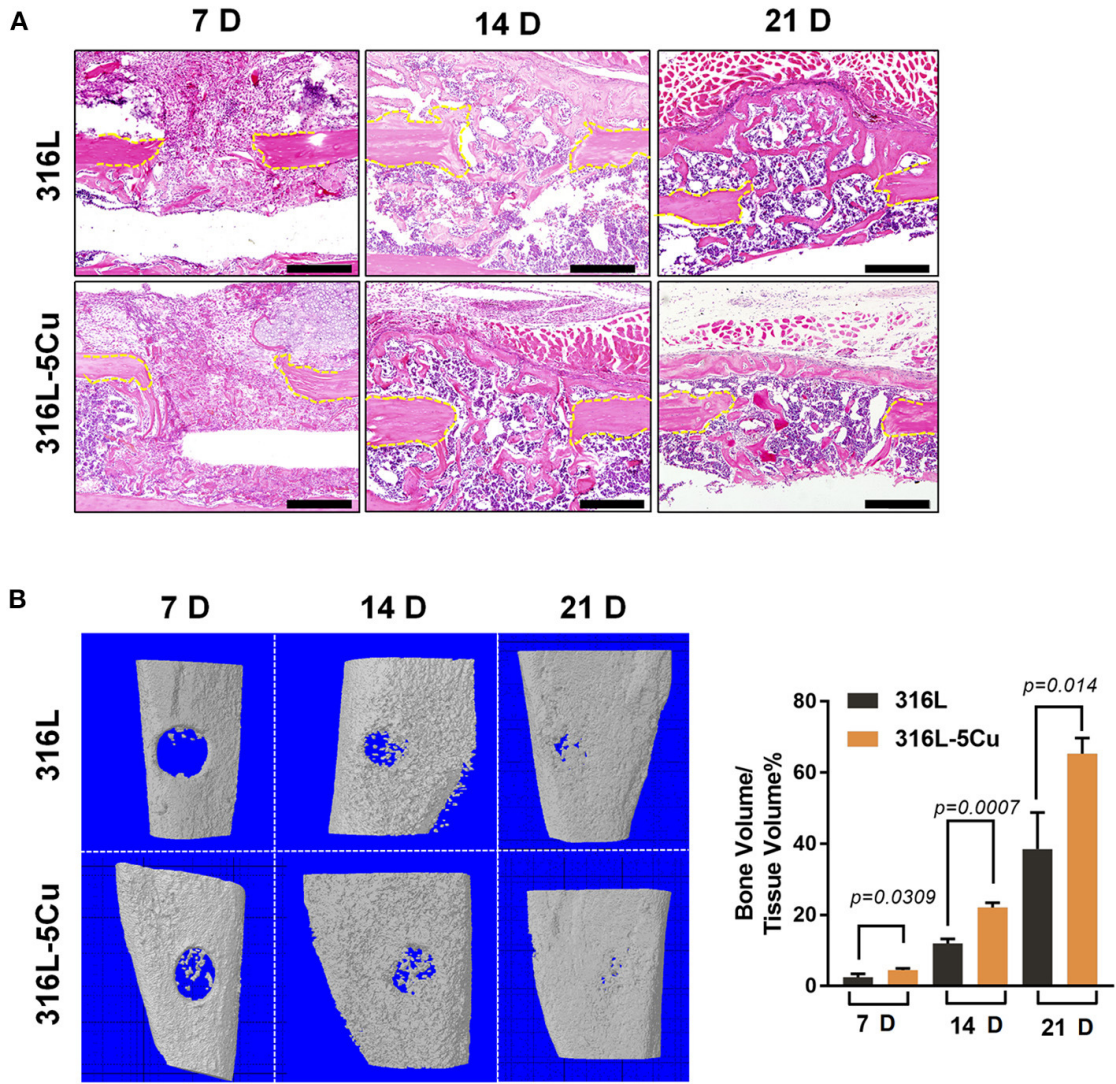
Copper-containing metal (CCM) accelerated callus formation in bone repair. **(A)** HandE-stained histological images of the tibia of 316L/316L−5Cu-inserted mice 7, 14, and 21 days after injury. Yellow dotted lines indicate the edge of the drill hole. Scale bar represents 200 μm. **(B)** Three-dimensional microcomputed tomography (μCT) images of the drill holes in 316L/316L−5Cu-inserted mice 7, 14, and 21 days after injury. The ratio of bone volume to tissue volume (BV/TV), representing bone formation in the drill holes, was calculated. Data were statistically analyzed by Student's *t*-test. Results are presented as mean ± SEM. *n* = 6 per time point per group.

To determine the role of M2a macrophages in bone regeneration, a tibia drilling hole injury model in C57BL/6 mice was established, and the closure degree of the drilling hole was measured and assessed by μCT and histomorphological procedures. The model was established by inserting 316L−5Cu/316L pins into the bone marrow cavity of the injured tibia as intramedullary nails. Formation of new cortical bone in the drilling hole was faster in the 316L−5Cu group than in the 316L group at days 14 and 21 (Figure 2A). The bone mineral content within the drill hole was almost 2-fold higher in the 316L−5Cu group compared with the 316L group at day 21 (Figure 2B). Using H&E staining, no difference was observed in the callus between the two groups at day 7, whereas the formation of cortical bone callus in the 316L−5Cu group was accelerated at the later stage. New and integral cortical bones were observed in the 316L−5Cu group, covering the top of the drilling hole on day 21 (Figure 2A).

**Figure 3:**
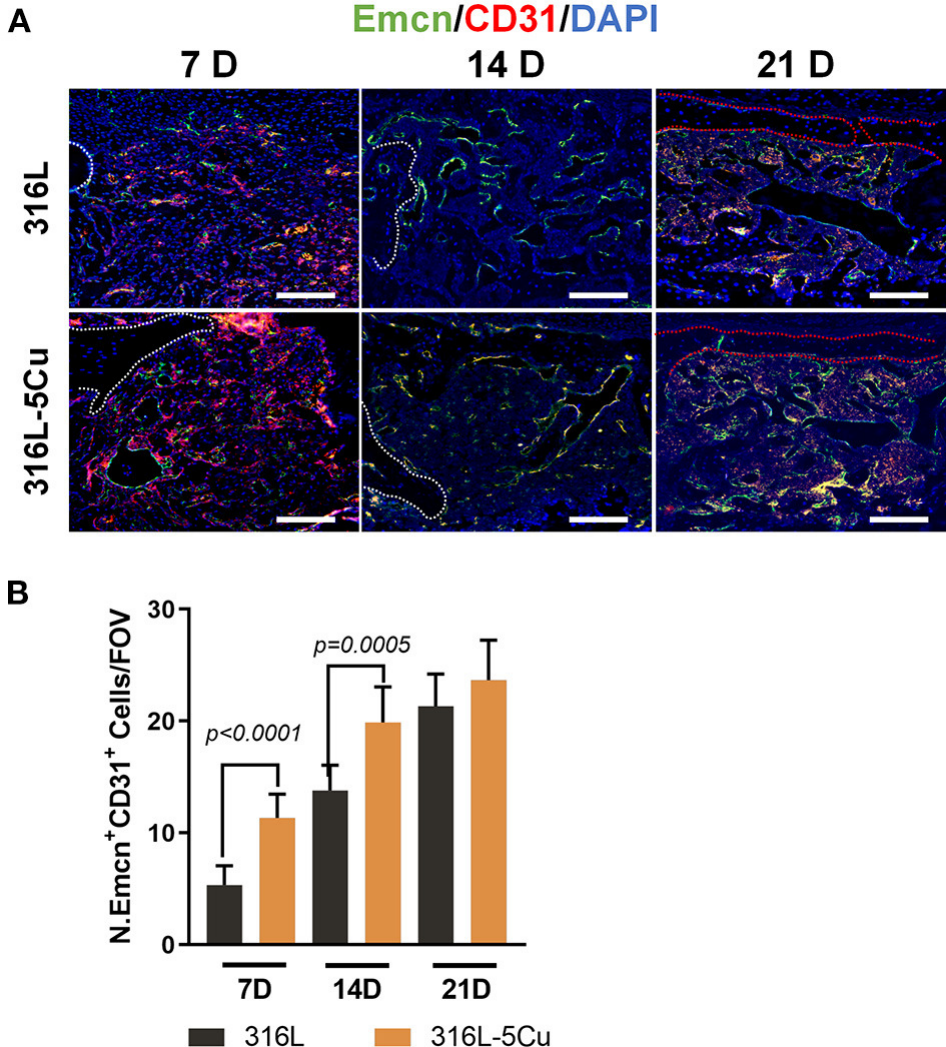
316L−5Cu significantly promoted the generation of type H vessels. **(A)** Representative images of double-immunofluorescence staining against EMCN (green) and CD31 (red). The merged yellow shows colocalization of EMCN and CD31. White dotted lines indicate the edge of the drill hole, and red dotted lines show the profile of the newly formed lamellar bone. Scale bar represents 100 μm. **(B)** Quantitative analysis of EMCN+ and CD31+ cells within the drill holes 7, 14, and 21 days after injury. Data were statistically analyzed by Student's *t*-test. Results are presented as mean ± SEM. *n* = 6 per time point per group.

Type H vessels have been reported to participate in bone fracture healing (Stefanowski et al., 2019). In the drilling hole injury model, expression levels of Emcn and CD31 were examined; these are markers of generation of type H vessels in immunofluorescence-stained tissue slices. Emcn+CD31+ immunofluorescence staining was observed on the bone callus; this was more intense from days 7 to 21 in the 316L−5Cu group, indicating the generation of type H vessels (Figures 3A,B). These signals were located particularly beneath the new cortical bone at day 21. These results show that 316L−5Cu significantly accelerated the formation of callus, accompanied by promotion of the generation of type H vessels.

### CCM Promotes Infiltration of CD206+M2a Macrophages into Callus During Bone Repair

**Figure 4:**
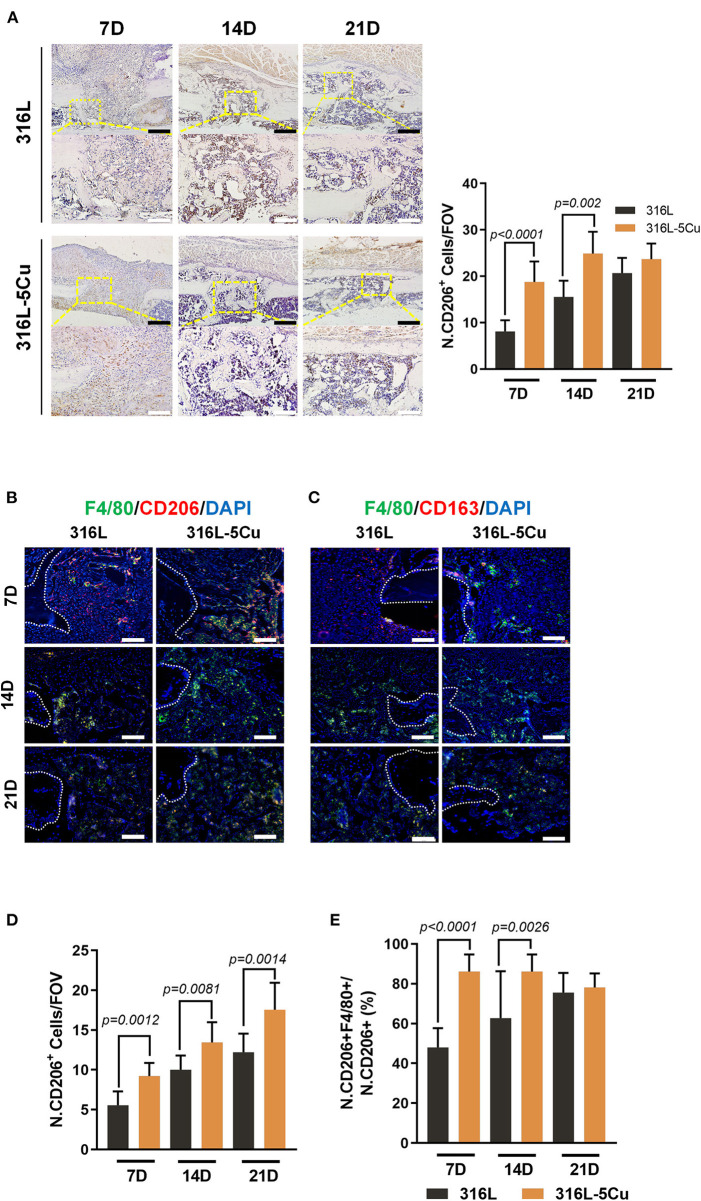
Copper-containing metal (CCM) promoted infiltration of CD206+M2a macrophages into callus during bone repair. **(A)** Representative images of immunohistochemical staining and quantification of numbers of CD206+ cells within the drill holes in 316L/316L−5Cu-inserted mice at days 7, 14, and 21. Black and white scale bars represent 100 and 50 μm. **(B)** Representative images of double-immunofluorescence staining against F4/80 (green) and CD206 (red). The merged yellow shows colocalization of F4/80 and CD206. **(C)** Representative images of immunofluorescence staining against F4/80 (green) and CD163 (red). The merged yellow shows colocalization of F4/80 and CD163. White dotted lines indicate the edges of the drill holes, and red dotted lines show the profile of the newly formed lamellar bone. White scale bar represents 100 μm. **(D)** Quantification of number of F4/80+ and CD206+ cells within the drill holes in 316L/316L−5Cu-inserted mice at days 7, 14, and 21. **(E)** Quantitative analysis of the fractions of F4/80+ and CD206+ cells within the drill holes in 316L/316L−5Cu-inserted mice at days 7, 14, and 21. All data were collected from at least three fields of view per sample and six samples per group. Data were statistically analyzed by Student's *t*-test. Results are presented as mean ± SEM.

Macrophages have been shown to infiltrate into the callus and periosteum during the healing of bone fractures; in particular, CD206+ M2 macrophages were primarily assembled in the periosteum, with very few present in the fracture gap at day 14 in a bone fracture model (Stefanowski et al., 2019). According to our results, CD206+ cells in both groups were located in the callus around the newly formed bone between the hole gaps. Furthermore, a significant increase in CD206+ cells was observed in the callus of the 316L−5Cu group (Figure 4A). We further detected CD163+ cells (also known as M2c macrophages) with an association with angiogenesis by immunohistochemistry and immunofluorescence. Few CD163+ cells were found during the process of bone regeneration by immunohistochemistry (data not published), but it was notable that a few 163+ (M2c) macrophages were located in hole gaps and in the bone marrow cavity, especially near the endosteum. There was no difference between the two groups (Figure 4C). We further detected the relationship between the CD206 and F4/80 immunofluorescence signals; coexpression of CD206 and F4/80 indicates M2a macrophages. From days 7 to 21, coexpression of CD206 and F4/80 exceeded 70% of the whole CD206 signal in the 316L−5Cu group and was significantly higher than 316L at days 7 and 14 (Figure 4E). The coexpression of the above two signals was also higher in the 316L−5Cu group compared with the 316L group during the bone regeneration process (Figure 4D). CD206+ M2a macrophages were found mainly in the callus of the drilling hole in the early stage (day 7) and around the surface of the newly formed bone (day 14). In the later stage, cells were observed beneath the new cortical bone (day 21), indicating a close relationship between M2a macrophages and newly formed cortical bone (Figure 4B).

### Copper Induced the Formation of Type H Vessels Activated by M2a-Macrophage-derived PDGF-BB

**Figure 5:**
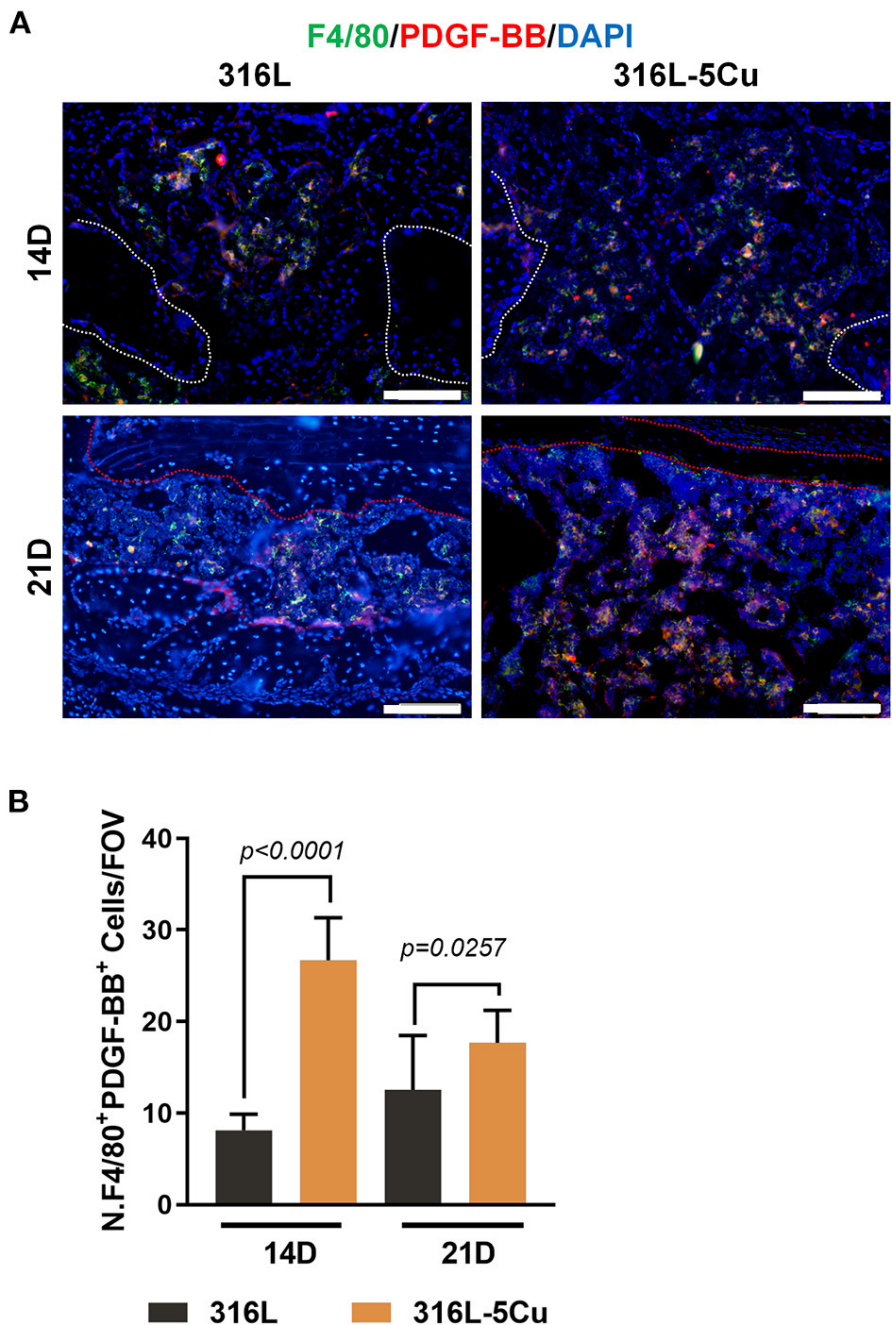
M2a macrophage-derived platelet-derived growth factor type BB (PDGF-BB) levels were elevated by 316L−5Cu. **(A)** Representative images of double-immunofluorescence staining against F4/80 (green) and PDGF-BB (red) at days 14 and 21. The merged yellow shows colocalization of F4/80 and PDGF-BB. Scale bars represent 100 μm. **(B)** Quantification of numbers of F4/80+ and PDGF-BB+ cells within drill holes in 316L/316L−5Cu inserted mice at days 14 and 21. Data were collected from at least three fields of view per sample and six samples per group. Data were statistically analyzed by Student's *t*-test. Results are presented as mean ± SEM.

PDGF-BB, which is secreted by M2a macrophages and TRAP– preosteoclasts, is critical for enhancing the formation of type H vessels and bone during bone modeling and remodeling (Xu et al., 2018). We examined PDGF-BB in callus (Figure 5A) and found that it showed significantly higher expression in the 316–5Cu group than in the 316L group at days 14 and 21 and that it was located in the F4/80+ macrophages beneath the new cortical bone (Figures 5A,B). At day 7, no difference in PDGF-BB expression in callus was found between the 316L−5Cu and 316L groups. These results indicate that the M2a macrophages secreted PDGF-BB in the revolution process of bone regeneration, which was promoted by 316–5Cu.

### High Levels of Mitochondrial-Derived Reactive Oxygen Species Might Enhance CCM-Promoted Expression of PDGF-BB by M2a Macrophages

**Figure 6:**
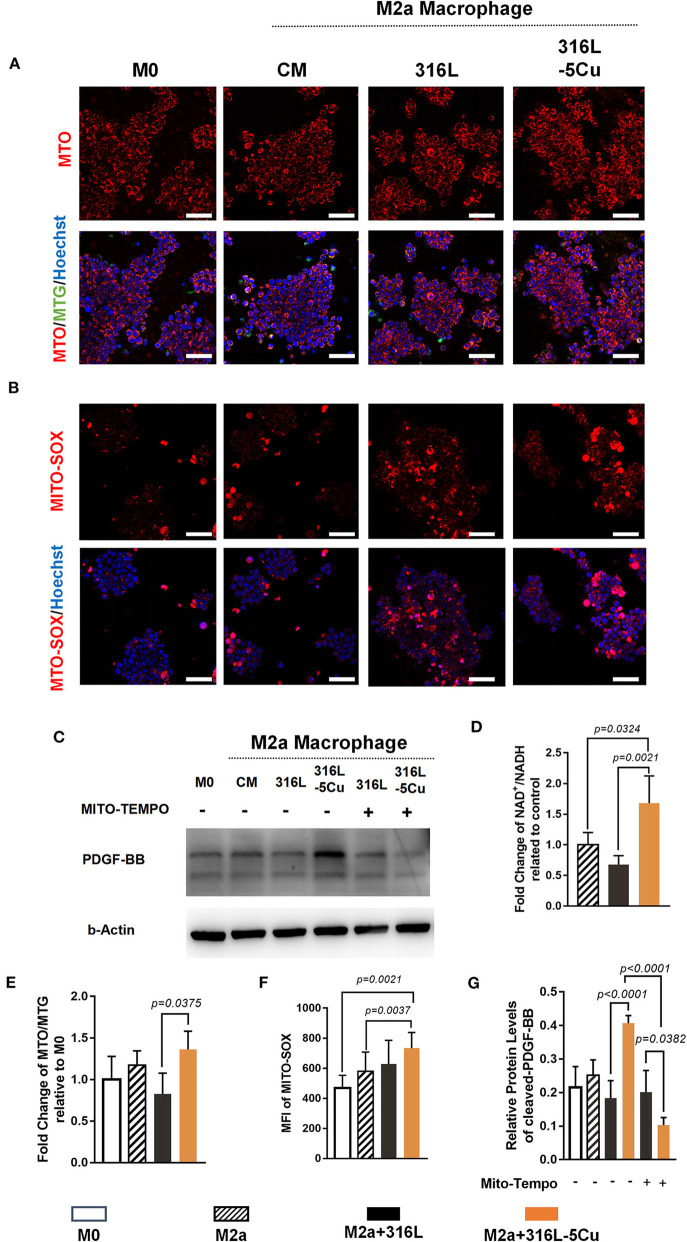
High levels of mitochondrial-derived reactive oxygen species (mtROS) could induce copper-containing metal (CCM)-promoted expression of platelet-derived growth factor type BB (PDGF-BB) from M2a macrophages. **(A)** Representative images of immunofluorescence of Mitotracker Orange (MTO), merge of MTO/Mitotracker Green (MTG)/Hoechst 33324 of M0 macrophages and M2a macrophages pretreated with vehicle (complete medium), 316L, and 316L−5Cu extract, respectively. **(B)** Representative images of immunofluorescence of MitoSOX, merge of MitoSOX/Hoechst 33324 of M0 and M2a macrophages pretreated with vehicle (complete medium), 316L, and 316L−5Cu extract, respectively. Scale bars in **(A,B)** represent 50 μm. **(C)** Western blotting was performed to examine levels of PDGF-BB in medium of M0 and M2a macrophages pretreated with vehicle, 316L, and 316L−5Cu. In addition, M2a macrophages pretreated with 316L and 316L−5Cu were further prestimulated with 50 nM Mito-TEMPO for 60 min before macrophage polarization. **(D)** Quantitative analysis of NADH/NAD+ in M2a macrophages pretreated with vehicle, 316L, and 316L−5Cu extract, respectively. Data were statistically analyzed by Student's *t* test. Results are presented as mean ± SEM. *n* = 5. **(E)** Quantification of fluorescence intensity of MTO/MTG based on the cell count, representing mitochondrial potential. **(F)** Quantification of mean fluorescence intensity (MFI) of MitoSOX based on the cell count, which was represented by mtROS. **(G)** Data were statistically analyzed by Student's *t*-test. Results are presented as mean ± SEM. *n* = 5.

Low-concentration copper preparations have been reported to have promising immunomodulatory potential and to contribute to oxidative stress in macrophages (Videla et al., 2003; Steinborn et al., 2017; Zhao and Zhao, 2019). Mitochondrial-derived reactive oxygen species (mtROS) are believed to derive mainly from NADH dehydrogenase when there is a high NADH/NAD+ ratio in the mitochondrial matrix. The inhibitory effect of copper on NADH dehydrogenase has been demonstrated in adenine–copper complexes and chelating 2-valent copper complexes (Hammud et al., 2008; Roy et al., 2015). To determine the role of CCM in inducing PDGF-BB expression by M2a macrophages, the oxidative stress caused by copper in macrophages was detected. According to our results, inhibition of NADH dehydrogenase activity occurred in M2a macrophages in the 316L−5Cu group, compared with 316L and control group (Figure 6D). In addition, levels of mitochondrial potential (Figures 6A,E) and mtROS (Figures 6B,F) were significantly elevated in IL4- and IL13-induced M2a macrophages by 316L−5Cu. Furthermore, the elevation of PDGF-BB expression in M2a macrophages induced by 316L−5Cu was found to be inhibited by a selective mtROS inhibitor (Mito-TEMPO) (Figures 6C,G).

## Discussion

Bone injury disrupts vessels, leading to reduced nutrient supply at the injury site. Vascularization is pivotal to the success of complete and scar-free bone regeneration (Lienau et al., 2009). After bone injury, type H vessels evolve in the bone formation region and persist throughout the entire bone regeneration process. The phenotype of type H endothelial cell structure, which has been described in endochondral long bone growth, is also reflected in bone regeneration (Kusumbe et al., 2014; Ramasamy et al., 2014; Filipowska et al., 2017; Stefanowski et al., 2019). Our findings showed that CCM inserted into the bone marrow resulted in a significant acceleration of callus formation by promoting the generation of type H vessels. Thus, it appeared that copper also took advantage of angiogenesis in bone regeneration.

Macrophages are a heterogeneous group of cells that carry out distinct functions in different tissues; they also lie on, or close to, the outer (abluminal) surface of blood vessels and perform several crucial activities at this interface between the tissue and blood. In addition to adaptive immunity, the inflammatory innate immune response is a major regulator of vascularization through the activity of different types of macrophages and the cytokines secreted (Lapenna et al., 2018). Macrophages exist on a spectrum of diverse phenotypes, from “classically activated” M1 to “alternatively activated” M2 macrophages (Murray et al., 2014). The M2a and M2c subsets that constitute M2 macrophages are typically considered to promote angiogenesis and tissue regeneration (Wang et al., 2017; Stefanowski et al., 2019). Lin's research on the incorporation of Cu^2+^ into bioactive glass ceramics showed that copper ions considerably induced the transition to M2 polarization of macrophages *in vitro*, possibly via activating the hypoxia-inducible factor (HIF) signaling pathway. However, M2 polarization was only verified *in vitro*, and its mechanism was unclear. Among the M2 macrophages involved in angiogenesis, CD206+M2a macrophages are known to specifically secrete high levels of PDGF-BB, whereas M2c macrophages secrete high levels of MMP9, an important protease involved in vascular remodeling (Wu et al., 2013; Spiller et al., 2014). In the present study, the subset of CD206+ macrophages was proved to infiltrate into the callus and was located beneath the newly formed type H vessels in the 316L−5Cu group, whereas few CD206+ macrophages were found in the 316L group. These results were consistent with those reported by Stefanowski et al. (2019). CD163+ macrophages have been proven to play a part in angiogenesis in the liver and brain but have not been reported to have a role in bone injury (Etzerodt and Moestrup, 2013; Nielsen et al., 2020). Few CD163+ macrophages were found to be involved in the process of fracture healing in either group, suggesting that the association between CD163+ and angiogenesis in bone injury is not strong. Our findings indicate that CCM accelerated the formation of callus in bone regeneration via activating the expression of PDGF-BB derived from M2a macrophages.

The immunoregulatory effect of intracellular copper metabolism has been examined in several studies (Videla et al., 2003; Steinborn et al., 2017; Zhao and Zhao, 2019). Deigendesch et al. (2018) found that depriving intracellular copper transporters of copper ions inhibited inflammasome activation in macrophages, thereby regulating the immune phenotype of macrophages. The present study found that mtROS were pivotal to the beneficial effect of CCM on bone repair. mt-ROS are believed to originate from proton leakage, which occurs mostly in complexes I and III (Angajala et al., 2018). Two copper-containing complexes were proved to inhibit the activity of mitochondrial complex I (NADH-UQ-reductase). In other work (not published), we found that bone infections treated with CCM also showed inhibition of NADH, resulting in an increase in mtROS. In this study, we found a similar upregulation of mtROS induced by copper in M2a macrophages, which promoted the expression of PDGF-BB. The increased PDGF-BB expression induced by copper was reduced by Mito-TEMPO, further confirming our results.

In our previous studies, the copper-containing stainless steel, 316L−5Cu, possessed a typical austenitic microstructure, have been proven to have the stable precipitating behavior *in vitro*, the release rate of copper ions was 5.079 ng/cm^2^/day over 28 days *in vitro* (Sun et al., 2016; Yang et al., 2017). Our other study showed that there was a significant increase in Cu^2+^ content in the bone calluses of the copper-containing stainless steel at 3, 6, and 9 weeks, but in serum. Over all, the Cu ions release from the steel was measured to be very tiny, <10 ppb/day (Wang et al., 2017).

Like the study of biochemical reagents, immunoregulation of copper-containing metal has become an important target in research on the use of biomaterials in bone disease (Dukhinova et al., 2019). In the past few years, the development direction of biomaterials engineering has shifted from “immune evasive” to “immune interactive,” with a focus on modulating the inflammatory response and promoting tissue regeneration (Taraballi et al., 2018). The effects on angiogenesis and osteogenesis of copper-containing biomaterials are continuously followed in bone diseases. Recently, the immunoregulatory effect of copper was reported in several studies (Lin et al., 2019; Liu et al., 2019). This study was the first to establish the links among immunoregulation, angiogenesis, and osteogenesis induced by copper-containing biomaterials in bone repair.

Taken together, these results demonstrate that formation of type H vessels was promoted during bone regeneration by the application of 316L−5Cu to drilling hole models in mice. CD206+ M2a macrophages in callus beneath the newly formed cortical bone were elevated by 316L−5Cu. The important crosstalk between M2a macrophages and vascularization was confirmed to be mediated by PDGF-BB. In addition, the promotion of PDGF-BB expression in M2a macrophages by CCM might have been induced by high levels of mtROS resulting from the inhibition of NADH dehydrogenase. Our findings underline the importance of CCM in the bone regeneration process by linking the innate immune response in bone repair to local angiogenesis.

## Data Availability Statement

The datasets presented in this study can be found in online repositories. The names of the repository/repositories and accession number(s) can be found in the article/supplementary material.

## Ethics Statement

The animal study was reviewed and approved by Ethics Committee of Nanfang Hospital of Southern Medical University.

## Author Contributions

BY, KY, and XZ designed the research. DX analyzed the data. LR and SZ prepared the materials. DX, JQ, XG, XH, XW, and HW performed the research. DX, XG, and KY wrote the paper. BY and XZ supervised the experiments and revised the manuscript. All authors contributed to the article and approved the submitted version.

## Conflict of Interest

The authors declare that the research was conducted in the absence of any commercial or financial relationships that could be construed as a potential conflict of interest.
